# Side Tracts

**Published:** 1889-04

**Authors:** 


### SIDE TRACKS.

During all my professional life I have, I trust, been traveling in one direction, and that is towards perfection. I have tried many roads that were inviting, some of which I soon found were leading me away from the goal at which I was aiming, and were at once abandoned. Especially during my early years did I attempt numerous short cuts and by-paths, that were guaranteed by enthusiastic confreres to land me at the desired haven in an almost incredibly short period of time; but invariably I found myself floundering in a swamp, if not warned in time by seeing my adviser, who had preceded me, stuck fast in the quagmire to which all short cuts inevitably lead. Experience has brought something of wisdom, and now when a half-balanced visionary urges upon me some gim-crack invention which entirely simplifies all dental operations, so that even a tyro is enabled to produce results to which long experience has not yet attained, I listen complacently, encourage his investigations, but stick to the old, well-worn road, until I see him come limping back, covered with the mud and bedraggled with the slime of the slough in which I was morally certain he would land.

I cannot remember half these by-paths into which I have been enticed, and into which I fear I have at times helped to inveigle others. One of the first was called plastic gold, and a beautifully shaded pathway it was until I had passed one or two milestones. Then the scene changed, and I was glad to get out of that gulf of despondency. I still occasionally hear the halloo of some one from that direction, who claims to have found solid ground in the morass; but I am satisfied with my experience, and feel certain that the neighborhood is treacherous footing.

Shredded or felted tin for awhile took me off my feet, but that I escaped from. Amalgam paths have opened on every side, and at the entrance of every one stood some loud-mouthed touter, vaunting the advantages, directness and abbreviations of his short line, and vowing that his way was pleasantness and all his paths were peace; but the unavoidable swamp stared me in the face when I took a good look ahead.

Then another side track opened, and that was called the oxy chloride-of-zinc path. I traveled this a good long distance, and got into the worst mess in which I have ever been entangled. How beautifully did a touter for this path display all its wonderful charms. He was an old and highly respected member of my profession, and he pictured the seductive road as the one specially opened for him, and incidentially for others, by angels with wing-feathers of extraordinary length and brilliancy, and they illuminated every foot of the way, causing it to shine with scintillations of their own glory. No more worry concerning those great rocks, exposed and even putrifying pulps. Saturate them with carbolic acid, place them in this new road and tenderly kiss them good-by; though wounded, sore and bleeding, the angels would look after the suffering ones and all would be sweetness and light. Well, these ministering spirits proved to be swamp angels, and they left me as usual, wallowing in the mire. I found that the great rocks must be surmounted by patient and toilsome effort. There was no royal road around them, and so, when long afterward another path called immediate root filling offered itself, and was persistently urged as the only short line to success, I remembered how I was bedraggled in a path of which this seemed almost the counterpart, and stuck to the highway.

Amputation of pulp was a little bridle path which opened out of the former one, and I think led to even a worse morass, but for a wonder, I did not get into that. Implantation is even now an inviting path, and I have tried the ground sufficiently to convince me that there is little of firm footing there. I might go on and enumerate a hundred other leafy by-paths which I have trod, or been urged to essay. Some of these seem alluring indeed, but someway I have almost invariably discovered that the one who was most importunate in demanding that I should step aside, usually had some proprietary interest in the line, or had at some vantage point erected his little toll-gate and demanded a contribution, either by way of license to travel, or through the sale of some help to locomotion which, he informed me, was essential to proper getting-on. I am getting to be an old bird, and rather a shy bird, and now when some voluble advocate of any short road to perfection, at society meetings or elsewhere, assails my ears with his auctioneer eloquence, I mentally ask him—"Let me see: where is *your* toll-gate. Is it at the very entrance of your patent path, or am I to encounter it a little further on?"

I am far from the assertion that there is nothing good in any of these little side-roads, but I do believe they are never fit for general travel. I do not regret that I tried any one of them. I only lament that I went so far at times as to lose sight of the main thoroughfare. Had I but strayed into them incidentally and gathered a few of the flowers that blossomed, carefully picking my way instead of blindly rushing along without taking due heed to my footsteps, I should not have brought up in the mire at their ending and been obliged painfully to retrace my footsteps until I laboriously found the old way again.

All my path of life in the past is indicated by land-marks, and each year is a milestone by which I can judge of the progress that I have made. I am happy in the belief that no two of them stand exactly together. The annual mile is of greatly varying length, and sometimes on looking back I can see that certain successive stones are a goodly distance apart. Others are nearer each other, but I do not believe that any one is not a fair distance in advance of its immediate predecessor. The early milestones are closest neighbors, because I did not then set myself down to the steady, plodding gait which alone tells in the race of life. But upon the whole I have made fair progress—not through swift running, but because I have honestly tried always to keep moving. I propose to continue this while I live, and if I could practice dentistry to the age of say two hundred and fifty, I think I might become reasonably skilful. I have no idea that I could even then become perfect, or accomplish that which so many of my confreres now claim to do—save one hundred, or even ninety per cent. of bad cases, exposed pulps, etc., but I should, I know, be far in advance of what I am to-day, for I believe that the annual milestones would grow further and further apart as the line lengthened and I learned more assiduously to shun the short-cuts and patent cross-lot paths to perfection.

Golden results are produced by golden means, and there is no excellence without great labor. No man who has attained to eminence in any walk in life ever got there through a side-cut. He went painfully and laboriously away around by the only sure path —the great highway of legitimate practice. Varney and Webb had no patent method of operating, but depended upon painstaking thoroughness, and I have never heard of any feather-minded seeker after by-paths and short lines to success who obtained results like theirs. The hardest lesson that young practitioners have to learn is to surmount obstacles instead of trying to go around them.

